# Is hyperprogressive disease a specific phenomenom of immunotherapy?

**DOI:** 10.37349/etat.2020.00027

**Published:** 2020-12-28

**Authors:** Marta Brambilla, Giuseppe Lo Russo, Roberto Ferrara, Sara Manglaviti, Marina Chiara Garassino, Mario Occhipinti

**Affiliations:** 1 Medical Oncology Department, Fondazione IRCCS Istituto Nazionale Tumori, 20133 Milan, Italy; 2 Medical Oncology Unit B, Policlinico Umberto I "Sapienza" University of Rome, 00161 Rome, Italy; Università Politecnica Marche, Italy

**Keywords:** Hyperprogressive disease, immunotherapy, chemotherapy-immunotherapy

## Abstract

Hyperprogressive disease (HPD) is a novel pattern of response during immunotherapy treatment. Several retrospective studies have evaluated its prevalence among various cancer types and, in particular, in non-small cell lung cancer patients, based on different definition criteria. If HPD is a just a typical phenomenon of immunotherapy is still an unsolved concern. This paper summarized the available data about HPD in other cancer treatments.

## Introduction

The advent of immune checkpoint inhibitors (ICIs) has completely changed the oncology clinical practice producing an increased survival benefit in various tumor types [1–5]. However, the use of ICIs has led to many therapeutic concerns for physicians, mainly due to the reported novel patterns of responses, such as pseudoprogression or hyperprogression. Hyperprogressive disease (HPD) is defined as an unexpected rapid tumor growth occurring in patients treated with immunotherapy. Approximately 3.8–29.4% of cancer patients and 13.8–37% of advanced non-small cell lung cancer (NSCLC) patients under anti-programmed cell death 1/ programmed death ligand 1 (PD-1/PD-L1) inhibitors, reported HPD [6]. It is still unclear if this phenomenon and its definition criteria are specific for ICIs and if HPD has been undervalued with conventional cytotoxic chemotherapy or target therapy.

## HPD criteria

HPD has been defined with different criteria among studies, so understanding the methodological diversity used to classify this phenomenon is essential to better understand the different results and to translate it into clinical practice.

One of the first definition of HPD included tumor growth rate (TGR). The TGR is able to define the kinetics of tumor growth before the start of treatment and its evolution after the beginning of ICIs. TGR is defined as a mathematical equation which considers the percentage of increase in tumor volume in a given time interval [7]. In this experience Champiat et al. [6], compared the tumor growth rate before (TGR B) and tumor growth rate during (TGR D) immunotherapy. HPD was defined as a progressive disease according to response evaluation criteria in solid tumors (RECIST) 1.1. at the first radiological evaluation with a 2-fold increase in the TGR ratio (TGR D/TGR B). The authors reported an inversely proportional correlation between the responses to immunotherapy and the TGR during the treatment period. The same criteria were used by Kanjanapan et al. [8] in their study. Ferrara et al. [9], defined HPD as a progression according to RECIST 1.1. at the first evaluation with an increase of more than 50% in TGR during ICI (TGR D) compared with TGR before ICI (TGR B) (TGR D-TGR B > 50%) [9]. Kato et al. [10], used a composite definition of HPD including size-clinical dependent and time dependent criteria: time to treatment failure (TTF) less than 2 months, an increase of more than 50% of the tumor load and an increase > 2 of the rate of progression during immunotherapy compared to the pre-immunotherapy period. Matos et al. [11], defined patients with HPD those who had a TTF < 2 months, a minimum increase in measurable lesions of 10 mm and an increase of at least 40% of the tumor burden or an increase of at least 20% associated with the appearance of new lesions. Saâda-Bouzid et al. [12], used tumor growth kinetics (TGK), a parameter essentially similar to TGR that takes into account the variation of the sum of the larger diameters of the target lesions per unit of time HPD was defined as a tumor growth kinetics ratio (TGKR) ≥ 2. TGKR is calculated as ratio of the slope of tumor growth pre-immunotherapy and the slope of tumor growth on-treatment. The TGKR does not bring intuitive information related to the difference in volume, since a doubling of the diameter means an increase of 8 times the volume [13]. Finally, in the manuscript by Lo Russo et al. [14], HPD was defined as a progression at the first radiological evaluation according to RECIST 1.1. associated with at least three of the following criteria: TTF < 2 months, ≥ 50% increase in the sum of target lesion diameters between baseline and first evaluation; the appearance of at least 2 new lesions in a previously involved organ; the spread of the disease to new organs; worsening of clinical conditions with an increase in PD according to Eastern Cooperative Oncology Group (ECOG) ≥ 2 during the first 2 months of treatment.

The great difference in the evaluation criteria for HPD reported in the different studies is the likely cause of the different incidences of HPD among cases series [6, 8–12, 14–16] (Table 1).

**Table 1.** List of the main criteria for HPD definition

**HPD definition**	**Drug**	**Type of tumor**	**Patients (*N*)**	**HPD (%)**	**Predictive factors**	**Reference**
PD RECIST and increase in TGR ≥ 2	mAb anti PD-1/PD-L1 (phase I)	Various	131	9%	Age ≥ 65 years	[6]
PD RECIST and ΔTGR > 50%	mAb anti PD-1/PD-L1	NSCLC	406	13.8%	> 2 metastatic sites	[9]
PD RECIST & increase in TGR ≥ 2	CKI and costimulatory molecules	Various	182	7%	Female	[8]
Increase in TGK ≥ 2	mAb anti PD-1/PD-L1	HNSCC	34	29%	Tumor relapse in RT	[12]
PD RECIST and 3 criteria among: 1) TTF < 2 months; 2) increase ≥ 50% in sum of target diameters; 3) ≥ 2 new lesions in already involved organs; 4) Mets in new organs; 5) PS ECOG ≥ 2	CKI	NSCLC	152	25.7%	Myeloids cell density and MPO in the tumor & low PD-L1	[14]
TTF < 2 months and increase in tumor burden ≥ 50% and increase in pace of growth ≥ 2	CKI and costimulatory molecules	Various	155	4%	EGFR, MDM2/4, DNMT3A	[10]
TTF < 2 months and increase ≥ 10 mm in mesurable lesions and increase ≥ 40% of tumor burden or > 20% with apperance of new lesions	CKI	Various	214	15%		[11]
TTF < 2 months and TGK ≥ 2 and increase in tumor volume ≥ 50%	CKI	NSCLC	135	13.1%	NLR > 4, LDH > UNL, STK11	[15]
PD RECIST and RECIST ≥ 50% and increase in TGR ≥ 2	CKI	Various		5 HPD patients	MDM2/4, EGFR	[16]

PD: progressive disease; PS: performance status; mAb: monoclonal antibody; CKI: cyclin-dependent kinase inhibitors; HNSCC: head and neck squamous cell carcinoma; RT: radiotherapy; MPO: myeloperoxidase; MDM2/4: Mouse double minute 2/4 homolog; DNMT3A: DNA methyltransferase 3 alpha; NLR: neutrophil lymphocyte ratio; LDH: lactate dehydrogenase; UNL: upper normal limit; STK11: Serine/threonine kinase 11

## HPD in other treatment: different characteristics of the same phenomenon?

HPD has been reported retrospectively in cancer patients under ICIs treatment, however we do not know if it is really a novel pattern of response exclusive of ICIs. Moreover, discontinuation of previous treatments, such as chemotherapy or targeted therapy, may also result in a “disease flare” that may simulate HPD. This phenomenon was defined for the first time by Chaft et al. [17], as a hospitalization or death attributable to disease progression after stopping the EGFR tyrosin kinase inhibitors (TKIs). They found out a disease flare in 14 among the 61 Caucasian patients evaluated (23%; 95% CI 14–35%). Nevertheless, it was not done a comparison of tumor kinetics growth before and after TKI discontinuation due to unavailable data. Chen et al. [18], reported a rate of disease flare of 8% in Asian patients after the cessation of EGFR-TKIs. Similar data were found also for patients discontinuing ALK-tyrosine kinase inhibitors or VEGFR-tyrosine kinase inhibitors [19, 20]. Moreover, cancer flare has been described for bone disease progression in NSCLC patients under EGFR-TKIs as an effect of an increased osteoblastic and healing activity and sign of therapeutic effect [21]. Acceleration of tumor growth during targeted agents (TAs) treatment has been described for RAF inhibitors and BRAF inhibitors [22–26]. Recently Matos et al. [27], evaluated the range of HPD in a cohort of advanced solid tumors treated in phase I trials with TAs in unapproved indications. From 119 patients who had progressive disease as best response, 26 (21.8%) were classified as HPD by RECIST criteria. No differences in overall survival (OS) were observed between HPD group (median OS 4.23 months; 95% CI 3.42–5.04) and non-HPD progressor group (median OS 5.7; 95% CI 4.99–6.4; HR 1.09, 95% CI 0.7–1.7; *P* = 0.70). This study has several limitations. It is a retrospective evaluation in which TAs, already approved for a clinical indication, were excluded; moreover, were included several tumor types and different kinds of target treatments and all patients were at umpteenth lines underlaying an intrinsic tumor resistance and aggressiveness. At least the use of RECIST 1.1 criteria may have overestimated HPD phenomenon in tumors with an aggressive intrinsic biology and underestimated it in patients with a rapid clinical deterioration without a confirmatory scan.

A rapid tumour cell proliferation was seen after induction chemotherapy in oropharyngeal cancer [28]. Nevertheless, to date there is only one study that evaluated the incidence of HPD between NSCLC patients under single agent chemotherapy. Using delta TGR Ferrara et al. [9], reported, in the 59 NSCLC patients included in their analysis only 3 HPD cases, with a median OS of 4.5 months (95% CI 2.5–6.5 months) at the landmark analysis at 6 weeks. Therefore, HPD was observed in 13.8% (56 of 406) of patients treated with PD-1/PD-L1 inhibitors compared with 5.1% (3 of 59) of patients treated with single-agent chemotherapy (taxanes 73%, pemetrexed 12%, vinorelbine 7%, and gemcitabine 8%). This study underlines that HPD is clearly more frequent in patients treated with immunotherapy but maybe not exclusive of these patients.

Even less is known about HPD presence in first line treatment with chemotherapy-immunotherapy. The efficacy of pembrolizumab in combination treatment was tested in two phase III studies in both squamous and non-squamous histotypes, respectively KEYNOTE 407 [29] and KEYNOTE 189 [30]. In the squamous histotype the addition of pembrolizumab to a platinum-based chemotherapy (associated with paclitaxel or nab-paclitaxel) has demonstrated to have a significant benefit over chemotherapy alone in progression free survival (PFS) and OS, regardless of PD-L1 status. Similarly, in patients with non-squamous histotype, the addition of pembrolizumab to a platinum and pemetrexed doublet showed a benefit in both OS and PFS compared to chemotherapy alone. In both studies the experimental treatment was not associated with an increased incidence of side effects or an accelerate progression of the disease. Even atezolizumab was evaluated in combination with first-line chemotherapy in patients with advanced lung cancer. In non-squamous histology, the association of atezolizumab has been evaluated in 3 phase III randomized trials: IMpower 150, 130 and 132 [31–33], respectively evaluating the addition of atezolizumab to a carboplatin-paclitaxel-bevacizumab, platinum salts-nab-paclitaxel, platinum salts-pemetrexed. All these studies showed a statistically significant advantage in their endpoints with the addition of immunotherapy to chemotherapy and no evidence of early deleterious effect in the combination arm. On the contrary, in squamous histology the addition of atezolizumab to a platinum-based doublet was evaluated in the IMpower 131 study and has not demonstrated a statistically significant benefit in terms of survival [33]. However, even in this study no detrimental effect was observed compared to chemotherapy alone.

Thus, understanding whether chemotherapy-immunotherapy may prevent HPD effect of ICI is still an unsolved question. Moreover, it is still unclear if the criteria used for single agent immunotherapy may be applied also in this setting. A recent report presented at the European Society for Medical Oncology (ESMO) 2020 compared the incidence of HPD between single agent ICI *vs.* combination therapy as front-line treatment in NSCLC. HPD occurred in up to 16% of PD-L1 TPS ≥ 50% NSCLC patients treated with first-line single-agent ICI compared to 6% in chemotherapy-immunotherapy. Furthermore, none of the HPD reported in the combination treatment was detected by dynamic indexes (TGR/TGK), suggesting that clinic-radiological features of HPD in this chemotherapy-immunotherapy may be different from the ICI single agent ones [34].

## Conclusions

The phenomenon of HPD has been highlighted with the advent of ICIs treatment. A possible explanation of HPD in patients treated with ICIs could lay in the enhancement of pro-tumorigenic effect of immune-system, such a switch to immunesuppressive tumor microenvironment by a stimulation of regulatory T cells via PD-L1 blockade [6]. Other possible mechanisms underlying HPD could be linked to tumor-infiltration by M2-like CD163^+^CD33^+^PD-L1^+^ clustered epithelioid macrophages [14] or to specific gene expression signatures [35].

Chemotherapy-immunotherapies treatment seems to lower the risk of experience HPD, suggesting that the use of combination treatments not inciting to immune-mechanisms may be a potential strategy to overcome HPD and maximize ICIs benefit. We have too little data to express an opinion regarding the possible presence of the HPD phenomenon in patients treated with chemotherapy or targeted therapy. Therefore, we need prospective studies to validate the hypotheses and to better understand HPD phenomenon.

## Abbreviations

ECOG: Eastern Cooperative Oncology Group
HPD: hyperprogressive disease
ICIs: immune checkpoint inhibitors
NSCLC: non-small cell lung cancer
OS: overall survival
PD-1: programmed cell death 1
PD-L1: programmed death ligand 1
PFS: progression free survival
RECIST: response evaluation criteria in solid tumors
TAs: targeted agents
TGK: tumor growth kinetics
TGKR: tumor growth kinetics ratio
TGR: tumor growth rate
TGR B: tumor growth rate before
TGR D: tumor growth rate during
TKIs: tyrosin kinase inhibitors
TTF: time to treatment failure

## Declarations

### Copyright

© The Authors 2020.
